# How many minors are participating in clinical research today? An estimate and important lessons learned

**DOI:** 10.1017/cts.2021.844

**Published:** 2021-09-06

**Authors:** Richard F. Ittenbach, Jeremy J. Corsmo, Alison D. Kissling, Arnold W. Strauss

**Affiliations:** 1 Pediatrics, Cincinnati Children’s Hospital Medical Center, Cincinnati, OH, USA; 2 University of Cincinnati College of Medicine, Cincinnati, OH, USA; 3 Edward L. Pratt Library, Cincinnati Children’s Hospital Medical Center, Cincinnati, OH, USA

**Keywords:** Pediatrics, regulatory science, bioethics, clinical research

## Abstract

Little is known about the number of minors enrolled in clinical research today. IRB administrators at leading pediatric medical centers were surveyed regarding studies with minors. Analyses were descriptive in nature with adaptive Bayesian bootstrap imputation used with missing data. Officials from 17/41 (41.5%) pediatric research centers responded: 74,204 active studies were estimated, 29,078 (39%) included minors, and 6574 (23%) were “more than minimal risk.” Minors accounted for 0.7–2.87M research subjects. Pediatric medicine desperately needs a more accurate and reliable reporting system for tracking the recruitment, retention, and involvement of minors in clinical research.

## Introduction

There are roughly ten times as many clinical trials involving adults as children in America today [1]. Yet, only two reports have estimated the number of families whose children have ever participated in clinical research, or would participate if risks were low enough [2,3]. This lack of depth in the literature exists despite important legislation intended to strengthen the scientific, therapeutic, and regulatory pathways to improve pediatric care (Pediatric Research Equity Act (2003) [4], FDA Modernization Action (1997) [5], Best Pharmaceuticals for Children Act [6], FDA Accelerated Approval Guidance) [7], and the importance of pediatric research to the broad fabric of medicine, in general [8]. The current research imperative emphasizing improved outcomes for all requires a deeper understanding of not only the population of pediatric research subjects but also the entire regulatory enterprise involving minors [9]. The purpose of this study was to answer a seemingly simple question, “How many minors are participating in clinical research today?” Secondary outcomes included related regulatory factors such as risk status of pediatric studies, an institution’s ability to distinguish between studies involving *only* minors and those with both minors and adults, and procedures used for gathering and reporting this information.

## Methods

Children’s hospitals, pediatrics departments, or combinations of both at academic medical centers, hereafter referred to as pediatric research institutions with $3.5M or more in FY 2018 NIH research funding served as the basis for this study. This funding line was selected as the qualifying threshold given the group’s $1.3B in funding accounting for ∼⅓ of the NIH’s total research funding (∼ $4.1B) for pediatric research in FY 2018, and that individual institutional funding dropped off precipitously at this point. A total of 44 institutions met this criterion (NIH RePORTER, https://reporter.nih.gov). Responses were received from 17 of 44 (41.5%) institutions, representing $778.8M (59.9%) of the $1.3B awarded to the group; 16 of 17 (94%) were affiliated with an academic medical center.

### Procedures

Human research protection program/Institutional Review Board (IRB) leaders (Director, Dean, Vice President, or Human Subjects Protections Officer) at the 44 qualifying institutions were identified and invited through email to complete a secure REDCap survey on December 28, 2018; three follow-up emails were sent, as necessary, to nonrespondents. The voluntary survey was conducted according to the Checklist for Reporting Results of Internet e-Surveys [10]. The 12-item survey consisted of three multipart questions assessing the number of (a) currently IRB approved, nonexempt human research studies; (b) studies enrolling minors only or minors/adults; and (c) studies with drugs/devices. Two questions regarding data accrual were also included. For purposes of this study, a minor was defined as someone 0–17.9 years of age (see line 1 of survey, Appendix A). The research activities included in this survey were exempt from ongoing IRB review (45CFR46.101(b)(2)), based on the pre-2018 common rule.

### Data Analysis

Data were primarily descriptive in nature (frequency counts, parametric and nonparametric measures of central tendency/variability); however, to account for missing data, a two-step imputation process was used beginning with an approximate Bayesian bootstrap imputation strategy with five donors per imputation for each missing observation. Once a complete data set was obtained for all responding institutions, a 1.5-fold correction was applied to the summed values to provide estimates for the 24 remaining institutions. Imputation was deemed appropriate given the nature of the study design, sample size, and need to add uncertainty to our estimates. Data were divided into two subsets, an unconditional part for which 14 of 85 (16%) values were missing and a conditional part for which 9 of 21 (43%) values required imputation. Content analysis was used for the subjective questions. Data were analyzed using SAS v9.4 TS Level 1M5 (SAS Corp, Cary, NC).

## Results

A total of 46,173 IRB-approved, nonexempt human subjects studies were reported by 16 of 17 responding institutions. With the aid of imputation for one institution and correction for the remaining 24 institutions, we estimate 74,204 active studies among leading pediatric research centers, 29,078 (39%) of which included minors. A total of 19,151 studies were classified as “minimal risk” for pediatric studies (IRB Sub-Part D determination), with 6574 considered ”more than minimal risk” and 5780 studies involved drugs or devices (8%).

Of the studies involving minors, only 3 of 17 institutions (17.6%) could distinguish between minors only studies (671 studies) and those involving both minors and adults (1761 studies); only one of three institutions could provide the number of minors (310,473) in their minors only studies. Of the 14 institutions that could not distinguish between the two types of studies, 7 reported 2.4M total research subjects. When estimating combined counts for the seven institutions failing to report data, we estimate 4.8M total research subjects participating in studies and 7.2M when scaling to the remaining institutions (Table 1).

**Table 1.** *Institutional characteristics by site* (*N* = 17)

Institution	Total active studies	Active studies with minors	Minimal risk	More than minimal risk	Drugs or devices	Number of studies minors (only)^a^	Number of subjects minors (only)^a^	Total number of subjects^b^
*Institutions with nondifferentiable databases*								
1	4194	1030	841	189	278			112,539
2	2774	926	772	154	215			**46,907**
3	2017	**853**	**713**	**141**	**156**			**498,709**
4	**3296**	**855**	**662**	**193**	**164**			**58,630**
7	3825	489	460	29	51			0
8	800	750	450	300	**285**			169,305
9	1820	1700	1140	560	560			1700
10	1500	180	30	150	31			1848
11	587	577	543	34	54			**23,247**
12	2708	460	104	356	50			**25,168**
13	2.97	1739	1259	480	**225**			2,097,316
14	3601	1393	976	417	391			**443,892**
16	2980	2736	**402**	**98**	**56**			**1,292,620**
17	6221	1890	1400	490	579			7757
*Institutions with differentiable databases*								
5	3150	483	442	41	74	186		
6	4796	1177	980	197	161	199		
15	2230	2147	1593	554	523	386	310,473	
Total (raw)	46,173	17,677	10,990	3951	2967	671	310,473	2,390,465
Total (with imputation)	49,469	19,385	12,767	4383	3853			4,779,638
Total (with correction)	74,204	29,078	19,151	6574	5780			7,169,457

*Note*. Names of reporting institutions were removed for confidentiality purposes. Bolded values represent imputed values as described in the Methods section of this paper

a denotes institutions that could distinguish between studies with minors only and those with minors and adults

b denotes institutions that could not distinguish between studies with minors only and studies with minors and adults. Because of the imputations of six cells, minimal risk studies + more than minimal risk studies may not always = number of active studies with minors

Institutional officials reported varied methods of gathering data. Data sources reported included electronic IRB systems (nine institutions), electronic medical records (one institution), and clinical trial management systems (five institutions). One institution reported manually gathering data from multiple sources (Table 2) and no institution reported having a single system for the requested information; none was able to respond to all questions.

**Table 2.** *Categorization of self-reported limitations in data acquisition and description of tracking systems used* (*N* = 15)

Institution #	Types of systems for gathering data			Limitations in software	Limitations in processes
	IRB^a^	CTMS^b^	EMR^c^		
1	✓			✓	✓
2	✓			✓	
3	✓			✓	
5		✓		✓	✓
6		✓		✓	✓
7	✓			✓	✓
8		✓		✓	
9	✓			✓	✓
10	✓			✓	✓
11		✓		✓	✓
12			✓		✓
13	✓			✓	✓
14	✓	✓		✓	✓
15	✓			✓	✓
17				✓	✓
Total	9	5	1	14	12

*Note*. Of the 17 participating institutions, two institutions (#4 and #16) did not respond; consequently, there are no data for these institutions in this chart

a IRB = electronic Institutional Review Board systems for managing IRB submissions or reviews

b CTMS = electronic Clinical Trial Management System or comparable electronic system used to manage day-to-day conduct of a clinical research study

c EMR = Electronic Medical Record system used within a hospital or healthcare setting

Officials were asked to describe limitations associated with reporting for this survey. Fifteen of the seventeen responding institutions reported limitations, which fell into one of two categories. The first category included software/IT-related limitations (*n* = 14), including inconsistent use of IT systems and an inability to report on its data. The second category consisted of internal processes, generally related to inconsistent or missing procedures used to collect/record information about subject enrollment (*n* = 12). Finally, eleven institutions reported limitations both in software/IT systems and in internal processes (Table 2).

## Discussion

The primary purpose of this study was to identify the total number of minors participating in clinical research today. Yet, other characteristics such as the number of active studies within an institution, the risk status of these studies, number of studies involving drugs or devices, and an institution’s ability to distinguish between minors only or combined minors/adults studies provide a reasonable context for interpreting this information.

A total of 74,204 studies were reported and estimated for the 41 institutions, of which 29,078 (39%) involved minors. While it is impossible to determine the accuracy of organizational-level data with respect to reporting standards for the risk status of studies involving minors, our 66% (19,151/29,078) rate of minimal risk studies involving minors is reasonably consistent with the Association for the Accreditation of Human Research Protection Program‘s (AAHRPP) rate of 68% for minimal risk studies undergoing expedited review for all age groups across all institutions as presented on page 22 of AAHRPP’s 2018 Metrics on Human Research Protection Program Performance, dated June 7, 2019 (https://admin.aahrpp.org/Website%20Documents/Metrics%20-%202018%20Metrics.pdf).

With respect to the actual number of minors participating in active clinical research, it was not possible to generate a specific and reliable estimate, as only 3 of 17 responding institutions reported the ability to distinguish minors from adults in studies that enrolled both minors and adults, and only one institution was able to report the number of minors participating in research studies (*n* = 310,473 minors), translating to 804 minors/study. With only one institution reporting a value, imputation for the others was not possible. A second institution initially reported number of enrolled minors, but follow-up queries to the institution found the value to be implausible and likely inaccurate (22.1M minors from 199 studies involving minors); consequently, the institution requested that the data be removed from the study database. A similar anomaly was identified for the total number of subjects (minor/adult), and it was removed from the database, as well.

With respect to institutions that could not distinguish studies enrolling minors only from those enrolling both minors and adults, a total of 7.2M subjects were reported and estimated. Obviously, substantial numbers of research participants were minors. Even if the number of minors enrolled in these studies ranged from 10% (717,000) to 40% (2.87M), this would constitute a substantial number of minors participating in research during the time of this survey. Therefore, we conservatively estimate as many as 2M minors participating in clinical research nationally at any point in time, which is markedly different from, and potentially much larger than, the 4 to 5M lifetime estimates provided by Davies et al. [2,3]. Sadly, even large, federally funded databases (https://clinicaltrials.gov) lack the ability to fully filter and enumerate the number of unique subjects per study across key age groups.

An evaluation of the comments regarding both the methods and limitations associated with responding to this survey yielded meaningful results. The predominant system used by 9 of 15 responding institutions was their electronic IRB system. The reliance on IRB systems may have been related to the survey being sent to IRB leaders, but may have also been related to these systems being a primary source for all human subjects research studies at an institution. The most common limitation related to the use of IRB systems for reporting is that IRB systems are generally designed to support the regulatory review and research oversight process. As such, consideration is not generally given to structuring data in a manner that facilitates reporting, often resulting in tedious, manual extraction efforts. The other commonly used data source, Clinical Trial Management Systems (CTMS), should be well suited to providing aggregate subject enrollment data. Our results support this assumption, with two of three institutions providing the most complete responses relying on CTMS solutions. The most common limitation pertaining to CTMS systems data was the inconsistent use of those systems, often resulting in incomplete data at the institutional level. The primary challenge for virtually all institutions was the lack of a source system for reporting aggregate enrollment data, including the ability to differentiate among groups within the same study.

There are two notable limitations to the methodology reported here. First, research protocols where institutions rely on external IRBs were intentionally excluded from this study, primarily because institutional officials and local IRB directors are unlikely to have the detailed information requested for studies when relying on an external IRB. Although AAHRPP’s 2018 metrics report supports that the majority of institutions that rely on external IRBs do so for less than 10% of all protocols, it is possible that this may result in an underestimate of the total number of studies reported and numbers of minors participating in such studies. Second, for the survey used in this study, minors were defined as children from 0 to 17.9 years of age. It is further noted that the age of majority in three US states (Alabama, Mississippi, and Nebraska) is not 18 years of age.

In conclusion, while it was not possible to estimate precisely the number of minors enrolled in clinical research from institutional reports, we were able to estimate conservatively an enrollment of 717,000–2.87M minors using combined minor/adult data. What is likely more important is the stark reality that even leading academic institutions seem to be unable to identify and report on the number of minors participating in research at their institutions. Such reality may not be surprising given the historically limited attention given to pediatric research by policy makers, federal funders, and regulatory oversight agencies that often, directly or indirectly, reinforce the long and outdated perspective that children are nothing more than “small adults.”

However, we know that this perspective does not have a sound scientific or biomedical basis and, with as many as two million minors participating in clinical research at any given time in the United States alone, the number of minors volunteering (or being volunteered) for clinical research is profound. Pediatric research institutions should be taking the lead in advocating for an increased focus on best practices for both increasing the number of minors participating in research and improving the practice of pediatric research, including more age-appropriate consenting practices and age-appropriate methods for directly engaging children in research and health-care settings. This improvement to practice must begin by simply knowing how many minors are actually participating in clinical research today. Without this foundational number, we have no way of asking more specific and more important questions like whether we are adequately representing all children, or if we are accurately and completely assessing other, related factors at play in pediatric research including ethnic-, racial-, or gender-related differences. While the survey data reported here are limited in scope, the take-away message is more poignant and compelling: leading pediatric institutions were unable to easily report the number of minors participating in clinical research at their institutions at the time of this survey. A more critical analysis is urgently needed to better understand and meet the research-related needs of American’s youngest and most vulnerable population.

## Appendix A Pediatric Protocols Study

[Investigator and Institution Deleted for Blinding Purposes]

The following survey is designed to estimate the number of minors (0 to 17.9 years of age) participating in IRB approved, non-exempt human subjects research nationwide. We understand that identifying the ‘number of children enrolled in IRB approved studies’ may be somewhat difficult to obtain. Because this is crucial to estimating potential for burden at the national level, we are asking for the best estimate possible.

In order to get this estimate, we are proposing an approach that includes extracting enrollment data from the most recent continuing review submitted for currently active studies conducted at your institution under your local IRB’s review—and targeting those studies that are enrolling a pediatric population (e.g. those that have a sub-part D determination). Thank you for your willingness to participate in this important pediatric study!

Please respond to each question or item relative to studies conducted at your institution under your local IRB review. In those cases where your IRB is responsible for a study conducted outside your institution, minors in those studies would not be included in your institutional response. Please note, this study is not limited to clinical trials or biomedical studies only. We are interested in all studies involving children at your institution.

1. Number of IRB approved, non-exempt human subject research studies, conducted at your institution ___
2. Of the number reported in (1) above, how many involve minors (0 to 17.9 years of age) as research participants (meaning your IRB has done a Sub-Part D review)? ___
  - Of the total number of active studies involving minors, how many are:
    1. Minimal risk: ___
    2. More than minimal risk: ___
  - Of the total number of active studies involving minors, how many involve drugs or devices ___
3. Of the number reported in (2) above, are you able to differentiate the studies that enroll minors only from the studies that enroll both minors and adults? Yes/No
  *If ‘Yes’ to (3) above, please respond to all of the following:*
  - For the total number of studies reported in (2) above:
    1. How many studies involve minors only? ___
      What is the total number of subjects participating in these studies? ___
    2. How many studies involve both minors and adults? ___
      What is the total number of subjects participating in these studies? ___
      *If ‘No’ to (3) above, please respond to the following:*
  - What is the total number of subjects participating in the studies reported in (2) above? ___
4. Briefly describe the method that was used to obtain these data (e.g., numbers were pulled from an enterprise CTMS system, from the most recent continuing review report to the IRB, or numbers were manually counted). [open field]
5. Please provide any known limitations or gaps in the numbers you reported. [open field]
6. Please provide the following:
  - Name of institution: ____________________
  - Is your institution affiliated with an academic institution? Yes/No
    *If ‘Yes,’ please specify which academic institution:* ____________________
  - Would you like a copy of the summary of this report when completed? Yes/No
